# Anchor threads can double the insect flight energy absorbed by spider orb webs

**DOI:** 10.1242/jeb.245123

**Published:** 2023-01-26

**Authors:** Sarah I. Han, Angela M. Alicea-Serrano, Todd A. Blackledge

**Affiliations:** The University of Akron, Biology Department, Akron, OH 44325, USA

**Keywords:** Spider web architecture, Web energy absorption, Web prey capture

## Abstract

To successfully capture flying insect prey, a spider's orb web must withstand the energy of impact without the silk breaking. In this study, we examined the anchor threads: the silk lines that anchor the main capture area of the web to the surrounding environment. These anchor threads can account for a large portion of the web, yet are usually excluded from experiments and simulations. We compared projectile capture and kinetic energy absorption between webs with and without access to anchor threads. Webs with anchor threads captured significantly more projectiles and absorbed significantly more energy than those with constrained anchors. This is likely because the anchor threads increase web compliance, resulting in webs with the ability to catch high-energy flying insects without breaking. Anchor threads are one example of how different types of web architecture expand the range of possible prey capture strategies by enabling the web to withstand greater impacts.

## Teaser

**Summary:** The anchor threads of spider orb webs allow greater movement of the web’s capture area, increasing the amount of absorbed kinetic energy and thus improving prey capture efficacy.

## INTRODUCTION

Spider orb webs combine the extraordinary material properties of diverse silks into an intricate architecture that helps spiders catch flying insect prey. For a successful capture, the orb web must stop and retain prey insects long enough for the spider to cross the web and capture them (Chacon and Eberhard, 1980; Eberhard, 1990; Opell, 1997). If the web is too rigid, an energetically flying insect will fracture the silk and pass through the web. If the web is too elastic, the flight energy of the insect will be returned, and the insect will rebound out of the web. An orb web therefore needs a ‘just right’ balance of strength and compliance to survive an impact and retain the insect (Lin et al., 1995; Swanson et al., 2006; Kelly et al., 2011; Yu et al., 2015).

**Fig. 1.**
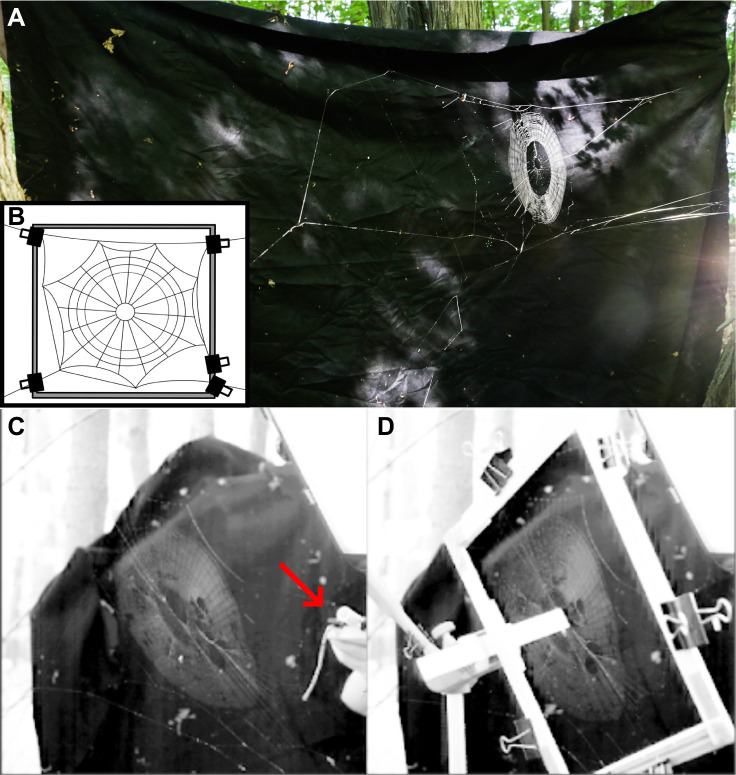
**A *Micrathena*** ***gracilis*** **web *in situ* and the two web testing states.** (A) A *M. gracilis* web spun between two trees. The bridge thread runs along the top of the web from tree to tree, and measures 163 cm, and the diameter of the capture area is only ∼12 cm. The web is in an untouched, natural state. Ten separate webs were used. (B) Schematic diagram of the constrained web showing the clamps and frame separating the capture area from the anchor threads. (C,D) Anchored (*n*=19; C) and constrained (*n*=16; D) testing states. The spring-loaded launcher is visible in C, indicated by an arrow.

A typical orb web consists of three different types of fibrous silk – a stiff, tough major ampullate ‘dragline’ silk and a stretchy flagelliform and aggregate ‘capture’ silk – built into a stereotyped architecture. The capture area is the circular region at the center of the web where a spiral array of capture silks can stick to insects. This capture spiral consists of extensible flagelliform silk fibers, coated with adhesive aggregate silk glue droplets, and is supported by an array of radial threads that spoke outwards from a central hub (Denny, 1976). This combination of strong and extensible silks keeps the web from breaking under impact while also absorbing the energy of the prey (Kelly et al., 2011). The capture area is connected to an outer frame of silk, and this frame is connected by anchor threads to the surrounding substrate, suspending the web midair. The top frame thread is often called the bridge thread (Zschokke, 1999), and anchors the web to the substrate (Fig. 1). Both the frame and anchor threads are composed of stiff dragline silk.

Many studies use either real or mathematically modelled webs to investigate how orb webs deal with the flight energy of insect prey (Denny, 1976; Harmer et al., 2015; Kelly et al., 2011; Lin et al., 1995; Sensenig et al., 2012, 2013; Tietsch et al., 2016; Zaera et al., 2014). These experiments suggest several mechanisms that webs use to absorb energy while catching prey. Energy is absorbed internally as the radial and capture lines strain under impact and dissipated externally through aerodynamic damping as the web moves through the air. The strong dragline radii absorb the majority of prey energy (Denny, 1976; Sensenig et al., 2012; Harmer et al., 2015; Yu et al., 2015). Capture silks absorb some energy from prey, but only a small amount (Yu et al., 2015). Some studies propose that the effect of aerodynamic drag is trivial (Sensenig et al., 2012), but other models disagree, claiming aerodynamic damping can have a major role in energy dissipation (Yu et al., 2015; Zaera et al., 2014) and prey capture (Lin et al., 1995). While these studies give significant insight into how orb webs deal with prey impact, they largely neglect a major component of the orb web that may have crucial implications for energy absorption – the anchor threads.

Anchor threads are the dragline silk lines that hold the web in position and connect it to the supporting substrate. Anchor threads can vary greatly in length among different spider species depending on the microhabitat preferences and other factors, though much is still unknown about what those factors are (Hesselberg, 2013; Mulder et al., 2021). In some webs, such as those of *Caerostris darwini* where the webs span rivers, anchor thread length can reach 10 times the diameter of the capture area, with the longest measuring up to 25 m (Gregorič et al., 2011a,b). Anchor threads are often reinforced with multiple draglines and are typically not consumed when orb webs are rebuilt daily. Thus, anchor threads can potentially comprise a large proportion of the total amount of silk in an orb web.

As the anchor threads are composed of the same strong dragline silk as the radii that absorb most of the prey energy, anchor threads could potentially contribute a large amount to total absorption of prey energy. Anchor threads are also the lines that attach the capture area to the substrate, and thus influence how much that whole area can move through the air, possibly affecting aerodynamic damping or the strain rate of threads in the capture area. Anchor threads may therefore play a significant, hugely understudied, role in the absorption of prey impact energy, and thus prey capture success.

The purpose of this study was to explore to what extent anchor threads affect prey capture through energy absorption. Overall, we wished to see how a web deals with prey impact with and without its anchor threads. More exactly, this study sought to estimate how much energy the anchor threads absorb when prey impacts a web, and whether that energy absorption improves the prey capture function of the web.

## MATERIALS AND METHODS

### Spider selection and experimental set-up

We chose the spined micrathena, *Micrathena gracilis* (Walckenaer 1805), as our study organism. *Micrathena gracilis* has some of the longest bridge and thus anchor threads relative to capture area size among local orb spiders, with lengths reaching almost 3 m (Uetz et al., 1978). For our experiments, we use the term anchor threads to refer to both bridge and anchor threads, as they serve the same function. Previous work looking at energy dissipation in orb webs has been done using artificial settings, either in the lab or *in silico*. As spinning webs of natural size would be difficult to achieve under laboratory confines, we found webs spun in the field and did all our testing *in situ*. This allowed us to test webs with anchor threads at their full length.

The webs of *M. gracilis* were located in a secondary growth forest at The University of Akron's field station in the Bath Nature Preserve in Bath, OH, USA. Webs were attached to a diversity of substrates: mostly between trees but also partially anchored to tall grass or the ground. *Micrathena gracilis* is a diurnal spider which builds a fresh capture area in its web every morning, so testing was done in the mornings to ensure that webs were freshly spun and whole. Adult female spiders were collected off the web, weighed and safely released in a nearby location before webs were tested. Anchor threads were measured across their entire span if possible, from substrate to substrate. If lines were not continuous, they were measured from substrate to the frame of the web.

To compare how the web absorbed prey impact energy with and without anchor threads, we used a physical clamp to temporarily constrain the anchor threads from moving or stretching during prey impact. We used binder clips to clamp a wooden frame onto the anchor threads outside of the capture area (Fig. 1), constraining the anchor threads without damaging the silk. We designated the framed webs that were separated from their anchor threads as the constrained state and the natural unframed webs as the anchored state. While under a constrained state, tests affected only the capture area within the frame. While under an anchored state, tests affected the entire web: capture area and anchor threads.

Testing was done using cylindrical wooden projectiles weighing ∼0.2 g as simulated prey, shot into the web perpendicular to the web plane using a spring-loaded projectile launcher at a distance of 60 cm from the web. Projectiles were shot by loading the projectile launcher at different tensions to achieve a range of impact energies. Each web was shot twice in total in each testing state and the order of which was first was randomized by flipping a coin. For example, for the anchored state first: anchored web 1, constrained 1, constrained 2, anchored 2.

Each projectile test was recorded using high-speed videography with a Photron SA4 camera (Photron, Tokyo, Japan) at 500 frames s^−1^, with the camera positioned at an angle to the web such that the camera was perpendicular to the projectile's movement, and the capture area could be visualized. This camera set-up allowed us to track the full motion of the projectile. A black cloth was placed behind each web to reduce background noise and provide contrast for the white silk strands. Two Fovitec S-900D LED (Fovitec, Hollywood, CA, USA) panel lights were used to light the webs. Videos were recorded in Photron Fastcam Viewer. Videos were analyzed using the motion-analysis software Proanalyst (Xcitex, Woburn, MA, USA). A 3D wire cube was used to calibrate projectile testing, and this image was used for perspective calibration within Proanalyst. The *x*–*y* coordinate data from each point tracked in Proanalyst were then analyzed in Microsoft Excel.

### Projectile energy absorption: anchored versus constrained

The projectile was tracked from before it contacted the web to just after it either broke through the web or was stopped by the web. Webs always broke in the capture area, never at the web–frame junction. Initial kinetic energy of projectile impact (KE_impact_) was determined from two frames prior to impact with the web. Final kinetic energy (KE_final_) was determined from two frames after the projectile broke through the web. If the projectile was stopped by the web, final kinetic energy was zero. The amount of energy absorbed by the web (KE_absorbed_) was determined as KE_absorbed_=KE_impact_−KE_final_.

The initial kinetic energies of the two web states were statistically compared with a *t*-test to see whether they were significantly different, and they were not (*t*_38_=−0.23; *P=*0.822). This showed us that the anchored and constrained states were undergoing similar energy impacts.

Each test was noted to be either a successful or failed prey capture. A successful capture meant that the web did not break and the projectile was tangled in the web at the end of its movement. A failed capture meant that the projectile broke through the web and ‘escaped’.

A total of 10 webs were tested. If the web became completely damaged by either testing or human error, trials were discontinued. We classified webs as completely damaged when over ∼50% of the web was destroyed. Constraining the web was challenging, causing webs to become too damaged on four occasions after the anchored state had already been tested. Because of this, we were not able to test four webs in the constrained state. In some tests, the web movement went out of the camera's frame, in which case KE_final_ and KE_absorbed_ could not be calculated for that test (*n*=2). In those cases, we merely noted KE_impact_ and whether it was a successful or failed capture.

### Maximum deflection and translocation

**Fig. 2.**
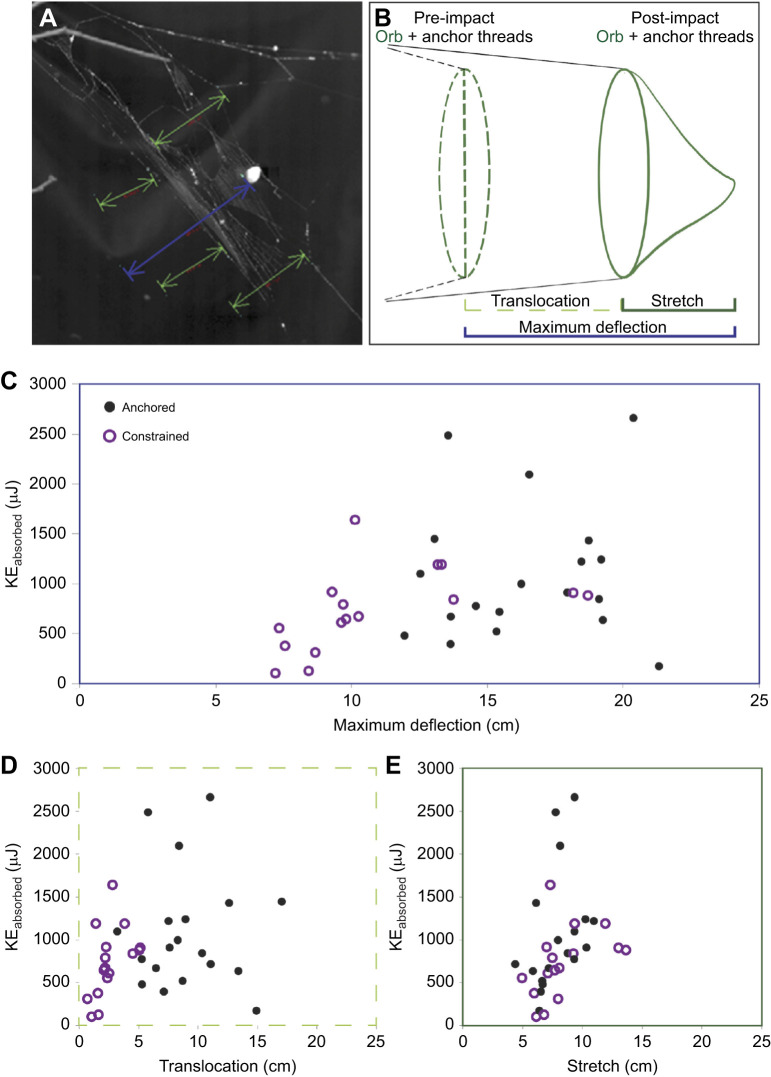
**Tracking projectile capture by individual webs and comparison of energy absorption between web states and movements.** Ten separate webs were used with *n*=19 anchored and *n*=16 constrained states. (A) Tracked areas of the web. The blue line indicates the center of the impact and measures maximum web deflection. The four green corner lines track the movement of the outer edge of the capture area and measure web translocation. (B) Diagram of pre-impact (dashed line) and post-impact (solid line) webs. Green denotes the capture area; black denotes the anchor threads. Three web movement stages were observed: maximum deflection is the maximum distance that the capture area both translocates and stretches around the projectile; translocation is the average distance that the capture area travels through space (an average of 4 vertex points) and stretch is the distance that the capture area stretches around the projectile. (C–E) Total kinetic energy (KE_absorbed_) absorbed by the entire web in the two states, plotted over the three web stages. (C) Maximum deflection is greater in anchored webs. (D) Translocation is greater in anchored webs. (E) Stretch is similar between the two web states. The presence of anchor threads allowed an average of 49.6% more maximum web deflection, driven mainly by translocation.

The web was analyzed for changes in position as the projectile contacted and moved through the web. Five landmarks were tracked within each web: the point in the capture area where the projectile impacted and four corner points at the junctions of the radial threads with the supporting frame at 90 deg radians relative to one another (Fig. 2A). We defined the maximum amount of deflection as the farthest point the capture area moved from its initial stationary position, which occurred where the projectile contacted the capture area. The four corner points defined web translocation, the distance that the entire capture area moved through 3D space. If more than two of the corner points moved out of frame or the maximum web deflection was out of frame, we excluded current and subsequent movement points. If only one or two corner points moved out of the field of view, then we simply averaged the remaining corner points. By subtracting web translocation from web deflection, we calculated web stretch as a measure of the maximum extension of threads within the capture area (Fig. 2B).

### Statistics

Stopping success was compared between anchored and constrained webs with a chi-square test. As we impacted each web 4 times in total (twice for each condition) with varying impact energies, we fitted mixed effects models to test whether the order of each impact and the KE_impact_ had any influence on absorbed energy, maximum deflection, translocation and stretch between anchored and constrained webs. For our models, we used the web number (1–10) as our random factor, order of impact and web state as our fixed factors, and KE_impact_ as a covariate. All statistical analyses were performed using Minitab software (Minitab, LLC, PA, USA).

## RESULTS AND DISCUSSION

We tested whether the anchor threads of orb webs play a significant role in stopping flying insect prey. Overall, allowing anchor threads to move and extend during impact resulted in 9-fold greater capture success and removed 58.4% more kinetic energy from simulated prey compared with orb webs where anchor thread motion was constrained.

**Fig. 3.**
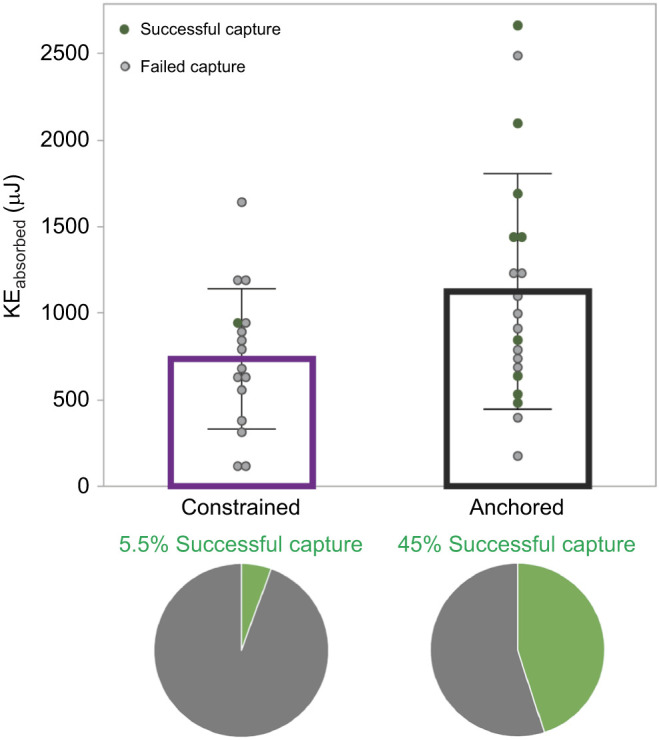
**KE_absorbed_ and percentage projectile capture by different web states in individual webs.** Ten separate webs were used with *n*=19 anchored and *n*=16 constrained states. KE_absorbed_ was significantly different in the two web states, with anchored webs absorbing significantly more energy. Circles represent capture success versus failure per trial. Bars represent mean and error bars represent s.d. Pie charts show that anchored webs were significantly better at catching projectiles than constrained webs (χ^2^, d.f.=1, *n*=38; *P*=0.006).

There was a clear difference in the rate of successful projectile capture (Fig. 3). Anchored webs, with a 45% successful capture rate, caught significantly more projectiles than constrained webs, which caught only 5.5% (χ^2^, d.f.=1, *n*=38, *P*=0.006).

The amount of kinetic energy absorbed by webs differed significantly between the anchored and constrained states (d.f.=1, *P*=0.039; Fig. 3). Anchored webs absorbed an average of 1164.8±734.6 μJ (mean±s.d.) while constrained webs absorbed an average of only 735±405.3 μJ. The order of impact did not have a significant influence (*P*=0.193) on the amount of absorbed energy, though the KE_impact_ did (*P*=0.001).

We looked at three different criteria of web movement: maximum deflection, translocation and stretch (Fig. 2). Maximum web deflection differed significantly between the two web states (d.f.=1, *P*<0.001). The constrained webs showed a maximum deflection of 10.93±3.6 cm and the anchored webs showed a maximum deflection of 16.36±2.9 cm, an average of 49.6% more than the constrained webs (Fig. 2C). KE_impact_ did not have a significant influence on maximum web deflection (*P*=0.692) in this case, but the order of impact did (*P*=0.004).

We separated maximum deflection into two component parts: translocation and stretch (Fig. 2D,E). Translocation showed the movement of the entire plane of the capture area, while stretch measured how much the radial and capture silks deformed out from that plane as a result of the projectile.

In anchored webs, translocation accounted for an average of 8.66±3.09 cm or 51.3% of the maximum web deflection, while in constrained webs, translocation contributed an average of 2.58±1.36 cm or 22.9% of maximum web deflection. In anchored webs, stretch contributed an average of 7.9±1.8 cm or 48.7% of maximum web deflection, while in constrained webs, stretch accounted for 8.36±2.51 cm or 77.1%. This showed that the two web states had similar amounts of stretch (d.f.=1, *P*=0.962), but that webs with anchor threads had significantly larger amounts of translocation (d.f.=1, *P*<0.001). Neither KE_impact_ (*P*=0.147) nor the order of impact (*P*=0.68) had any effect on translocation, but both KE_impact_ (*P*=0.018) and order of impact (*P*=0.032) were significant factors in stretch.

While the capture areas of anchored webs moved through almost 50% more space than those of constrained webs, the threads within the capture areas stretched to the same degree in anchored and constrained webs. This suggests that there is a critical stress after which the capture area of the webs will break that is unaffected by anchor threads. Additionally, we saw that the order of impact influenced the amount of stretch, likely because the capture area became more compliant after becoming damaged. The anchor threads make the whole orb web structurally more compliant so that more work to remove energy from prey can be done before reaching that critical stress. This work could be done through tensile deformation of the anchor threads themselves. Alternatively, aerodynamic damping can occur as thin threads move through air (Lin et al., 1995; Jiang and Nayeb-Hashemi, 2020). While the importance of aerodynamic damping relative to tensile deformation of silk is debated (Sensenig et al., 2012), both mechanisms would be facilitated by the increased distances that orb webs extend when supported by anchor threads.

Though our study focused on how anchor threads affected how the webs dealt with impact, we did not test specifically for variations in anchor length. Because of methodological difficulties during field work, we were unable to challenge all webs with similar impact energies. As a result, we were unable to correlate anchor length with energy absorption (*r*^2^=0.14). However, our study clearly demonstrates that some anchor length is better than none (Fig. 2). Future studies would include fully evaluating how anchor length changes energy absorption.

The substrate on which the web is built can also influence energy absorption. *Micrathena gracilis* normally builds webs in large open spaces between trees or branches (Biere and Uetz, 1981), but sometimes builds on more flexible substrates. One web in our study was anchored to long stalks of grass. This web, which had the shortest anchor threads, was subjected to some of our highest impact energies in the anchored states (2096 and 3652 μJ). Despite this, it was able to absorb large amounts of the imparted energy (100% and 68%, respectively). In these anchored trials, translocation contributed 51% and 43% to total web deflection. We suspect that the flexible grass stems acted as an extension of the anchor threads, enabling the web to absorb large amounts of energy without the need for longer anchor threads. Substrate effects on the kinematics of web capture also warrant future investigation.

Another source of variation may be due to how projectiles impacted the web. As in nature, our projectiles struck the web in different spots. This meant that the number of flagelliform or radial threads struck changed for each impact. Our projectile was also cylindrical, meaning the applied stress varied depending on the face that impacted the web. Lastly, the number of dragline strands in the anchor threads could cause variation. The capture area of *M. gracilis* webs is replaced daily; however, the anchor threads may remain for days (Biere and Uetz, 1981; Uetz and Biere, 1980). Although we did not examine the number of dragline strands in anchor threads in our study, we have seen that they are highly variable. Possibly, differences in the volume of major ampullate silk may contribute to the amount of variation.

As anchor threads have the potential to improve prey capture so dramatically, why don't all orb spiders invest in longer anchor threads? Some silks may be tough enough to stop prey sufficiently well on their own, rendering additional silk length unnecessary. Additionally, niche partitioning may lead to different spider species building webs in different microhabitats that help define the space available for constructing webs (Olive, 1980; Blackledge et al., 2003). Some species, such as *Eustala illicita*, build very short anchors, possibly to help deal with high-density population sites (Hesselberg, 2013). One particularly spectacular example of how microhabitat correlates with anchor thread length is Darwin's bark spider. The long anchor threads of *Caerostris darwini* anchor its web over rivers, and this spider, renowned for constructing the largest known webs with the toughest known dragline silk (Agnarsson et al., 2010; Gregorič et al., 2011a,b), builds a sparser web than other similarly sized webs (Sensenig et al., 2010). It is possible that *C. darwini* can build these sparser webs because the spider is using both better and more silk. By using tough silk, which can absorb large amounts of energy, and long anchor threads, which can both stretch and allow web translocation, *C. darwini's* web should be highly optimized for capturing its prey of medium/large insects (Gregorič et al., 2011a,b).

Our results demonstrate that the additional silk contained in anchor threads increases the overall movement and compliance of the capture areas of orb webs, potentially doubling the absorbed energy and leading to more effective prey capture. Our finding that the anchor threads cause such a huge improvement in energy absorption and stopping success demonstrates the critical importance of studying webs as complete systems. By looking at how the whole web responds to impact energy, we can better understand how silk material properties and web architecture change web compliance, and how that benefits prey capture. Through this we may gain a deeper insight into how nature creates flexible structures through differing arrangements of tough materials.
